# Association between GRIK1 rs363598 and intergenic rs360932 variants and susceptibility to autism spectrum disorders in Egyptian children

**DOI:** 10.1186/s12887-025-06229-9

**Published:** 2025-12-01

**Authors:** Heba Bassiony, Ahmed Baiomy, Doaa Ahmed, Nesma M. Elaraby, Tamer H. A. Ammar, Engy A. Ashaat

**Affiliations:** 1 Zoology Department, Faculty of Science, Cairo University, Giza, 12613 Egypt; 2 Cell Biology, Histology and Genetics, Faculty of Science, Cairo University, Giza, 12613 Egypt; 3 Medical Molecular Genetics Department, Human Genetics and Genome Research Institute, National Research Centre (NRC), Giza, 12622 Egypt; 4 Clinical Genetics Department, Human Genetics and Genome Research Institute, National Research Centre (NRC), Cairo, 12622 Egypt

**Keywords:** Tetra-primer, ARMS-PCR, Variations, ASD, And GRIK1 gene

## Abstract

**Background:**

Autism spectrum disorder (ASD) is a genetically inherited, complex neuropsychiatric developmental condition that impacts a person's ability to learn, interact, and communicate. ASD is currently classified as a heterogeneous disorder, given that the pathophysiology of ASD is yet unknown. The GRIK gene family (GRIK1, GRIK2, GRIK3, GRIK4, and GRIK5) has genetic variants associated with many psychiatric illnesses including; depression, obsessive–compulsive disorder, and autism. The present study is the first to determine the possible association of GRIK1 rs363598 and intergenic rs360932 variants with susceptibility to ASD in Egyptian children and to correlate these variants with different parameters.

**Subject and methods:**

One hundred children with ASD and one hundred volunteer healthy children served as control group were enrolled. Clinical parameters were measured. The genotyping method was performed in all children using the Tetra-primer Amplification Refractory Mutation System-Polymerase Chain Reaction (ARMS-PCR) technique.

**Results:**

ASD cases were mainly associated with males (77%) than females (23%) (*p* = 0.014) with lower IQ than the healthy group. The mean score on the Childhood Autism Rating Scale (CARS) was 39.40 ± 6.25. Interestingly, the genotype of the rs360932 SNP showed a significant difference in distribution between the ASD patients and the healthy control group (OR = 2.84, 95% CI = 1.29–6.35, *P* = 0.008); the genotype (AG) was substantially associated (90%) with ASD. Conversely, no discernible variation was seen in the distribution of the rs363598 SNP (OR = 3.19, 95% CI = 0.83–12.1, *P* = 0.07). Furthermore, the distribution of alleles for both variations did not differ significantly between the group of people with ASD and the healthy group.

**Conclusions:**

Egyptian children's increased risk of developing ASD is highly correlated with the rs360932 variation. Future research on other SNPs and genes linked to ASD may benefit from this study's increased chances of examining these topics.

## Introduction

Autism spectrum disorder (ASD) is a neurodevelopmental disease based on the Diagnostic and Statistical Manual of Mental Disorders, fifth edition (DSM-5) classifications and the International Classification of Diseases, Tenth Revision (ICD-10) criteria, characterized by persistent deficiencies in social interaction and communication [1, 2]. ASD symptoms include repetitive behaviors/restricted interests, loss of eye contact, extreme fear, difficulty reacting to names, and a lack of pretend play and interaction, the degree of severity in autism by using the Children's Autism Rating Scale (CARS) which varies from one child to another [3, 4].

Typically, children with ASD can be diagnosed between the ages of 18 and 24 months [5]. According to Zeidan et al. 2022, 1/100 children are globally diagnosed with ASD [6]. In 2018, the Centers for Disease Control and Prevention (CDC) reported that around 1 in 59 children were diagnosed with ASD [7]. In Egypt, a community-based survey estimated that 3.3% (95% CI: 3.1–3.5%) of children aged 2–12 years are at high risk for ASD [8], while the prevalence of confirmed diagnoses was 1.1% (95% CI: 1.0–1.2%) [9]. It was reported that the condition is typically influenced by both environmental and additional genetic factors that affect the developing brain [10]. It is difficult to determine the underlying genetic factors precisely. According to previous studies, autistic features could be associated with fragile X syndrome, Rett syndrome, tuberous sclerosis (10–20%), structural chromosomal abnormalities (3%), and metabolic disorders (10–20%) [11]. However, Genetic variations, including single-nucleotide polymorphisms (SNPs), are primarily responsible for approximately around 50% of ASD incidence [12].

A Genome-Wide Association Study has been carried out in this area to help determine the genes linked to a particular illness and look for SNPs [13]. Thus far, various candidate genes including MECP2, RELN, CNTNAP2, OXTR, and GABA receptor subunits have been identified in autistic individuals and repeatedly evaluated in multiple studies with large sample sizes [14, 15]. To date, few studies have been conducted on glutamate receptor (GRIK) genes [15]. The GRIK gene family (GRIK1, GRIK2, GRIK3, GRIK4, and GRIK5) has genetic variants that contribute to various illnesses, including Huntington's disease [16], Parkinson's disease [17], autism [18], and schizophrenia [19].

The GRIK1 gene encodes specific ionotropic glutamate receptor (GluR) subunits, which are classified as a subtype of the kainate receptor. RNA editing and alternative splicing regulate the receptor assembly and intracellular trafficking of GluR. These receptors play a regular synaptic role in promoting excitatory neurotransmission [20]. Besides, GRIK1 variants have been determined for their associations with several diseases, such as alcohol dependence [21], juvenile absence epilepsy [22], and the effects of topiramate on binge drinking [23].

Interestingly, Genome-Wide Association Study on SNPs suggested that the glutamate receptor, ionotropic, kainate 1 (GRIK1) gene's rs363598 and intergenic rs360932 SNPs are correlated with autism spectrum disorder (ASD) [24–26]. Moreover, the GRIK1 gene rs363598 T > C and the intergenic rs360932 G > A were linked to the development of ASD in a prior study on Bangladeshi children [15]. While in Egypt, the association between these two SNPs and ASD is unknown, it is crucial to investigate the association between these two SNPs and ASD in Egyptian children. rs363598 SNP is oriented on human chromosome 21 at position 29,640,839. The SNP rs363598 is an exonic non-synonymous SNP that changes the amino acid sequence of the protein encoded by the gene. This can alter the protein's structure, function, or stability, potentially impacting its biological role [27]. rs363598 has been studied in various neurodevelopmental disorders, including ASD. Specifically, it is associated with an increased risk of ASD in some populations. The C allele of rs363598 is associated with increased risk for ASD and other conditions like GRIK, major depressive disorder, bipolar disorder, and schizophrenia [28]. The intergenic rs360932 SNP is located on chromosome 4 in humans at position 151,987,017. It's situated roughly between SLC25A26 and PLSCR1 genes. Previous research recorded an association between the 'A' allele and ASD, major depressive disorder, bipolar disorder, and attention deficit-hyperactivity disorder [29, 30]. Nevertheless, the polymorphic data of GRIK1 derived from several investigations lack complete consistency and conclusiveness. These results suggest that the GRIK1 gene may be primarily responsible for cognitive disorders.

Consequently, it was intriguing to employ the Tetra-primer ARMS-PCR approach in our case–control study to investigate whether the GRIK1 gene's rs363598 and intergenic rs360932 SNPs are associated with an increased risk of ASD in Egyptian children.

## Materials and methods

The present case–control study's methodology included two groups of Egyptian children; the first group consisted of one hundred autistic children, and the second group consisted of one hundred *age- and sex-matched children* with normal neurodevelopmental milestones, *who served as the* healthy control group, clinically free from any neuropsychiatric diseases, with good mentality (IQ range between 90 and 120), and with good performance in school.

ASD children were recruited for the present study after initial diagnosis, which was performed at the Clinical Genetics Clinic of the National Research Centre (NRC), Cairo, Egypt, between 2021 and 2023. The autistic children were diagnosed according to DSM-5 classifications and ICD-10 criteria. *The degree of severity was further assessed using* The Children's Autism Rating Scale (CARS). Permission to use the CARS was obtained from the copyright holders prior to data collection. We employed the validated Arabic version of the CARS, which has been translated and culturally adapted for use in Arabic-speaking populations, including Egyptian children. This version has demonstrated good reliability and validity for identifying ASD in Middle Eastern and North African populations [31, 32]. In the present study, the CARS was administered and scored by trained clinicians specializing in childhood neurodevelopmental disorders, ensuring standardized administration and inter-rater agreement. A clinical examination was performed on all children with autism, including a review of consanguinity and details of their history. *A comprehensive history was also collected, covering* delivery history, family history (including parental consanguinity and similarly affected family members), clinical examination of all body systems, gastrointestinal test (GIT) symptoms (such as constipation and abdominal pain), and sleep disorders, which are commonly associated symptoms with ASD.

Investigations, including electroencephalogram (EEG) and intelligence quotient (IQ) assessment, as well as CARS scores, were performed on all patients [33, 34]. It should be noted that autism-specific assessments (e.g., CARS scoring, severity classification, and behavioral/clinical features) were collected only in the ASD group, as these measures are not applicable to typically developing children. These variables were used exclusively to stratify and characterize autistic children and to explore potential correlations with genetic variants, whereas the genetic analyses were performed in both groups to enable valid case–control comparisons.

CARS was used to assess each autistic child to identify the severity of ASD. ASD severity is categorized into three levels: mild (level 1), moderate (level 2), and severe (level 3), based on the amount of support an individual needs to function in daily life. These levels reflect the extent of social communication impairments and repetitive behaviours and interests. The CARS scores range from 15 to 60, with higher scores indicating more severe symptoms. A CARS score of 15–29.5 means the individual is likely in the non-autistic range; 30–36.5 suggests mild to moderate autism; and 37–60 indicates severe autism. Children with autism were diagnosed with mild, moderate, and severe ASD depending on their total CARS scores [31, 35].

All children underwent a *comprehensive* medical history assessment, including sex, age, demographic data, family history of similar illnesses, history of consanguinity in parents, and three-generation pedigree analysis.

### Sample size

The study enrolled 100 cases and 100 controls based on both feasibility and prior precedent in autism genetics research. To further support adequacy, we conducted a post-hoc power analysis using PASS 2024 software [36] (NCSS, Kaysville, UT, USA). Under a dominant genetic model, we assumed a control minor allele frequency (MAF) of ~ 0.40, consistent with previous reports of common ASD-related variants [37, 38], and an odds ratio (OR) of 2.5, which lies within the range reported in autism case–control studies from Egyptian and regional cohorts [39]. This translates to an expected carrier proportion of 0.64 in controls and 0.816 in cases under Hardy–Weinberg equilibrium.

With 100 cases and 100 controls and two-sided α = 0.05, the PASS Chi-Square test (uncorrected) for two proportions yielded power ≈ 82%, indicating that our study is sufficiently powered to detect moderate-to-large genetic effects.

The study protocol followed the Helsinki Declaration and was approved by the ethical committee of the National Research Centre (No: 4421022023). Written informed consent was obtained from all guardians, with information disclosure conducted to ensure comprehension, consistent with local recommendations emphasizing patient and guardian understanding as the core purpose of consent [40].

### DNA extraction

Three milliliters of whole blood were drawn from each child. Genomic DNA was extracted by using the PAXgene Blood DNA Kit (Qiagen, Germany). The purity and concentration of the extracted DNA were measured at wavelengths of 260 nm and 260/280 nm, respectively, using the NanoDrop 2000 (Thermo Scientific, USA). Pure DNA has a measured ratio of ~ 1.8 at wavelength 260/280. The integrity of DNA was confirmed by electrophoresis on 1% agarose gel stained with ethidium bromide. DNA samples were stored at − 80 °C for further analysis.

### Genotyping of GRIK1 gene rs363598 and intergenic rs360932 variants

Genotyping of the GRIK1 gene rs363598 and intergenic rs360932 variants were analyzed in DNA samples of all children employing the Tetra-primer Amplification Refractory Mutation System-Polymerase Chain Reaction Technique (Tetra-primer ARMS-PCR) [41]. In this method, the amplification is achieved using a multiplex reaction involving two sets of outer primers: the forward and reverse outer (FO, RO) primers to amplify distinct amplicons of the gene of interest, independent of the allele present at the SNP site. The forward and reverse inner primers (FI, RI) produce allele-specific amplicons with RO and FO primers, respectively. There will be variations in the sizes of these amplifiers. Where the two outer primers ensure the PCR efficiency and the gene specificity, the inner-outer combination (FO/RI, FI/RO) ensures the allele specificity.

Using the tetra-primer ARMS-PCR approach, a single reaction combines two inner SNP-specific primers and two outer primers, including purposeful mismatches at the 3' end of the inner primers at position − 2 to enhance the specificity of amplification. Utilizing (http://primer1.soton.ac.uk/primer1.html), a two-specific set of primers was developed, and the BLAST tool (https://www.ncbi.nlm.nih.gov/tools/primer-blast/index.cgi?GROUP_TARGET=on) was used to assess the specificity. The optimal primers and annealing temperature for the GRIK1 gene rs363598 and intergenic rs360932 variants are listed in Table 1.

**Table 1:** Primers and annealing temperature used for the GRIK1 gene polymorphism genotyping

SNPs	Primer sequences	Annealing temperature (°C)
*GRIK1* rs363598	FO: AACAGTGTAAATGGGACAGAATTCA RO: AAACTTGCCTTTCAATGTGGAG FI: TCAGAAAATGGCAGTTAAAGAACAG RI: TTAATTGCTCTGGGTGCTTTAAAT	57
rs360932	FO: TTAAATATGCCTAAATGTGGCTTG RO: ATACCAGGAATTTCAAACTGGC FI: ACTGCAGGGAAAAGGAAGC RI: TGCCTAATAGAAAAACATCACATCA	56

The amplification reaction was conducted in one tube and a single step of PCR. GoTaq master mix, nuclease-free water, MgCl_2_, (FI, RI) primers, and (FO, RO) primers at the proper concentrations were added to create the PCR working master mix. For a 120 μL PCR solution, the volumes of the outer and inner primers were 7.2 μL and 12 μL, respectively. Then, the total volume of solution (120 μL) was divided into 12 PCR tubes, with 10 μL used for each reaction. Next, the 10 μL master mix solution was mixed with the necessary quantity of DNA from each of the individual cases and controls. This PCR reaction volume was kept constant for both the controls and the cases.

Initial denaturation for 5 min at 94 ⁰C, followed by 35 cycles of PCR cycling, consists of denaturation for 30 s at 94 ⁰C, annealing for 30 s at a suitable temperature (Table 1), extension for 30 s at 72 ⁰C, with a final extension for 7 min at 72 ⁰C. The acquired PCR product was electrophoresed on a 3% agarose gel to separate it, which was stained with ethidium bromide in opposition to the DNA marker of 100–1500 bp. The separated bands were examined under UV light and photographed.

### Statistical analysis

Statistical analyses were performed using IBM SPSS Statistics version 22 (SPSS Inc., Chicago, IL, USA). Descriptive statistics were used to summarize the data. Continuous variables were expressed as mean ± standard deviation (SD), and categorical variables as frequencies and percentages. The Shapiro–Wilk test was used to assess the normality of continuous variables. Since the data were normally distributed (*P* > 0.05), comparisons between groups were performed using the parametric independent samples t-test. The chi-square (χ^2^) test was used to analyze differences in categorical variables. Allele frequencies were calculated using gene counting. Logistic regression analysis was conducted to estimate odds ratios (ORs) and 95% confidence intervals (CIs) for genetic associations. A *P*-value of < 0.05 was considered statistically significant.

## Results

### Demographic variables for cases of ASD and healthy controls

Table 2 provided a demonstration of demographic traits of the participating patients and healthy controls. It was discovered that 23% of ASD patients were female, while 77% of instances included men, indicating that there is a higher correlation between ASD and men (*P* = 0.014).

**Table 2:** General demographic characteristics of the study groups

	ASD Patients (*N* = 100)	Healthy control (*N* = 100)	*P* Value
Gender (%)			
Male	77 (77.0%)	61 (61.0%)	0. 014*
Female	23 (23.0%)	39 (39.0%)	
Age _(years)_			
(Mean ± SD)	7.22 ± 3.97	8.1 ± 2.6	0.06
IQ score range	65–80	90–120	0.05*
CARS			
(Mean ± SD)	39.40 ± 6.25	-	-
Degree of disease			
Mild	41 (41.0%)	-	0.05*
Moderate	31 (31.0%)		
Severe	28 (28.0%)		
Consanguinity			
-ve	71 (71.0%)	100%	-
+ ve	29 (29.0%)	0%	
Similarly affected family members			
-ve	58 (58.0%)	-	-
+ ve	42 (42.0%)		
Complicated delivery history			
-ve	74 (74.0%)	-	-
+ ve	26 (26.0%)		
EEG changes			
-ve	58 (58.0%)	-	-
+ ve	42 (42.0%)		
GIT disorders			
-ve	45 (45.0%)	-	-
+ ve	55 (55.0%)		

Comparisons were performed using an independent samples *t*-test

Data are presented as means ± SDs, otherwise –; not available

*CARS* Childhood Autism Rating Scale, *GIT* Gastrointestinal, *EGG* Electroencephalogram

-Ve = negative (not found in the patients), + Ve = positive, (found in the patients). The degree of disease was classified according to the CARS Score; 30–36.5 indicates mild to moderate, and 37–60 indicates a severe degree of disease

^*^Significant difference at *P* value ≤ 0.05

**Fig. 1:**
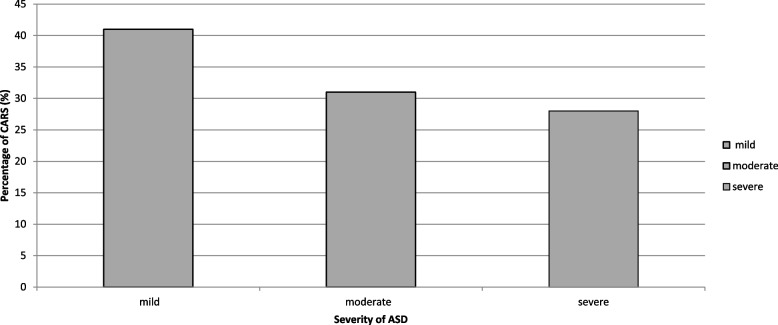
The percentage of the degree of CARs for the ASD group

The age distribution between the patients and the healthy controls was not statistically significant. IQ scores were significantly higher in the healthy group than in the ASD group (*P* = 0.05). The mean score for the Childhood Autism Rating Scale (CARS) was 39.40 ± 6.25, indicating a statistically significant difference. According to CARS degree, most children had mild ASD (41%), while moderate and severe ASD were observed in 31% and 28% of the cases, respectively (*P* = 0.05). The score distribution for the CARS was displayed in Fig. 1; there were 48% of the ASD children had affected families with psychological disease. As well, only 26% of ASD cases have a history of complicated delivery history. The electroencephalogram (EEG) showed abnormalities in 42% of ASD cases, while the gastrointestinal test (GIT) revealed abnormalities in 55% of ASD cases.

### Genotyping of GRIK1 gene rs363598 and intergenic rs360932 polymorphisms in ASD cases and healthy controls using Tetra-primer ARMS-PCR

**Fig. 2:**
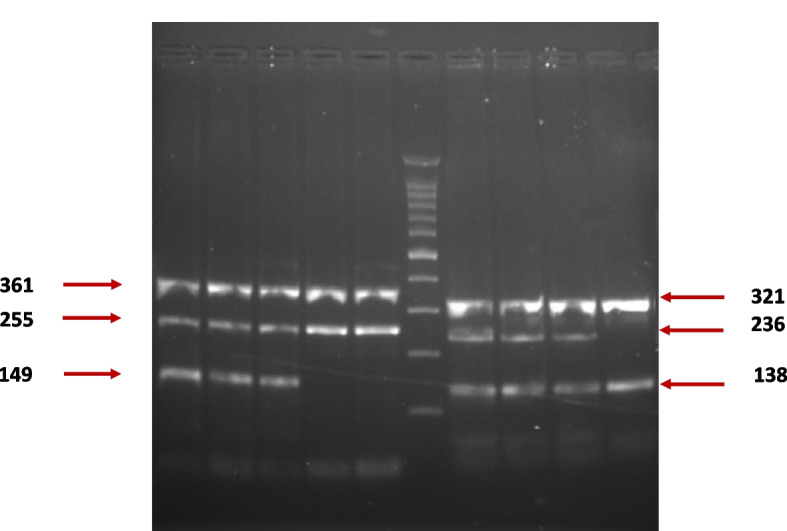
Image of gel electrophoresis representative of the separation of ten samples of Tetra-primer ARMS-PCR products for GRIK1 gene rs363598 and intergenic rs360932 polymorphisms. 2% agarose gel was used for ethidium bromide staining and electrophoresis to demonstrate the following: Lanes 1–3 present the TC genotype for rs363598, lanes 4 and 5 show the TT genotype for rs363598, lane 6 presents a 100 base pair molecular weight marker, lanes 7–9 show the rs360932 GA genotype, and finally lane 10 shows the rs360932 GG genotype

The genotype variations from the two selected SNPs were visualized directly using standard agarose gel electrophoresis, as seen in Fig. 2.

#### Association of rs360932 and rs363598 variants with ASD

**Fig. 3:**
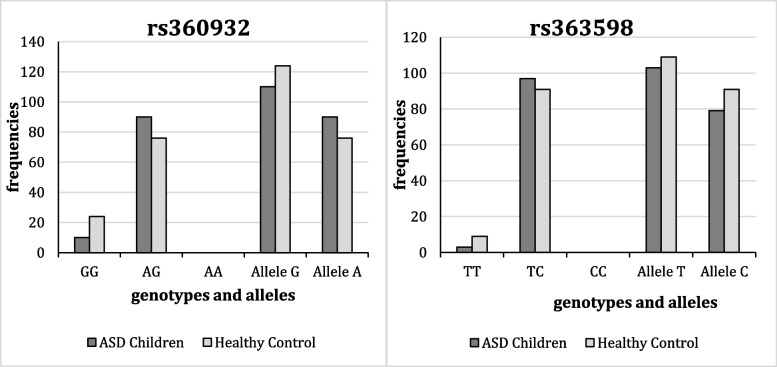
Genotyping and allele frequencies for GRIK1 gene rs363598 and intergenic rs360932

The polymorphism of two variants in the GRIK1 gene, rs360932 and intergenic rs363598, was estimated in autistic patients and healthy controls. The frequencies of each allele and genotype are summarized in Table 3 and Fig. 3.

**Table 3:** Genotype distribution and allele frequency between ASD patients and healthy control groups

Groups	ASD Patients *N* (%) = 100	Healthy Control *N* (%) = 100	OR (95% CI)	*P* Value
SNP				
rs360932				
Genotypes				
GG	10 (10.0%)	24 (24.0%)	Ref	0.008*
AG	90 (90.0%)	76 (76.0%)	2.84 (1.29–6.35)	
AA	0 (0%)	0 (0%)	-	
Alleles				
G	110 (55.0%)	124 (62.0%)	Ref	0.155
A	90 (45.0%)	76 (38.0%)	0.749 (0.503–1.116)	
rs363598				
Genotypes				
TT	3 (3.0%)	9 (9.0%)	Ref	0.07
TC	97 (97.0%)	91 (91.0%)	3.19 (0.83–12.1)	
CC	0 (0%)	0 (0%)	-	
Alleles				
T	103 (51. 5%)	109 (54.5%)	Ref	0.548
C	97 (48.5%)	91 (45.5%)	0.88 (0.538–1.31)	

Comparison was carried out by using the chi-square (χ2) test

*SNP* Single-nucleotide polymorphism, *OR* Odds ratio, *CI* Confidence interval

Data are expressed as n (%): number of subjects (percentage)

*Significant difference at *P* value ≤ 0.05

#### Association of rs360932 and rs363598 variants with ASD

The polymorphism of two variants in the GRIK1 gene, rs360932 and intergenic rs363598, was estimated in autistic patients and healthy controls. The frequencies of each allele and genotype are summarized in Table 3 and Fig. 3. Statistically significant variation in the genotype distribution of the rs360932 SNP was detected between the ASD patients and the healthy control group (OR = 2.84, 95% CI = 1.29–6.35, *P* = 0.008). The incidence of the AG genotype was significantly higher in both groups compared to the GG and AA genotypes, which were not detected in either group. Furthermore, compared to the control group of children (76.0%), the ASD patients had a greater percentage of AG genotype (90.0%). In contrast, the healthy control group had a larger number of GG genotypes (24%) than did the ASD patients (10%). Regarding the allele frequency, the incidence of allele G was higher in both groups than that of allele A; however, this difference was not statistically significant (OR = 0.749, 95% CI = 0.503–1.116, *P* = 0.155).

Additionally, subgroup comparisons were conducted between G-carriers (GG + AG) and AA, as well as A-carriers (AA + AG) vs GG. These analyses did not reveal any statistically significant differences between the studied groups.

On the contrary, in the case of the rs363598 SNP, it was found that the incidence of the TC genotype is higher in both ASD children and the control children (97.0% and 91%, respectively) than that of the TT genotype (3.0% and 9.0%, respectively). At the same time, there was no detection of the CC genotype in either of the studied groups. However, this difference in the genotype distribution was not statistically significant (OR = 3.19, 95% CI = 0.83–12.1, *P* = 0.07). Similarly, the percentage of the T allele was higher in both the ASD children and the control children than the percentage of C allele, although this difference was not statistically significant (OR = 0.88, 95% CI = 0.53–1.31, *P* = 0.548).

Those findings indicate that, in the AG genotype, the rs360932 variation is substantially associated with an increased risk of developing ASD. In contrast, neither genotype shows a significant association with the other mutation, rs363598, and the development of ASD.

#### Correlation between the demographic and the clinical features of ASD patients and genotype variants GRIK1 gene rs363598 and intergenic rs360932

The genotype polymorphisms of the two variants, GRIK1 gene rs363598 and intergenic rs360932, were statistically correlated with the demographic and clinical features of the autistic patients; the frequencies were summarized in Table 4.

**Table 4:** Correlation between the demographic and clinical features of ASD patients with the genotype distribution of gene variants

	rs360932 Genotype Frequency (%)			*P* value	rs363598 Genotype Frequency (%)			*P* value
	GG *N* = 10	AG *N* = 90	AA *N* = 0		TT *N* = 3	TC *N* = 97	CC *N* = 0	
Age	7.9 ± 5.01	7.14 ± 3.8	-	0.542	3.97 ± 2.7	7.32 ± 3.9	-	0.143
Gender								
Male	4(40.0%)	73(81.11%)	-	0.00*	3(100%)	74(76.28%)	-	0.33
Female	6(60.0%)	17(18.89%)			0	23(23.7%)		
CARS	45.9 ± 6.9	38.66 ± 5.7	-	0.001*	37.17 ± 62.7	39.46 ± 6.93	-	0.53
Degree of disease								
Mild	2 (20.0%)	39(43.33%)			2(66.6%)	39(40.2%)	-	0.5
Moderate	1 (10.0%)	30 (33.3)%	-	0.01*	1(3.33%)	30(30.9%)		
Severe	7 (70.0%)	21(23.33%)			-	28(28.8%)		
Affected								
_ -ve_	6(60.0%)	52(57.78%)	-	0.89	0	58(58.0%)	-	0.39
_ +ve_	4(40.0%)	38(42.22%)			3(100%)	39(40.2%)		
Consanguinity								
_ -ve_	6(60.0%)	65 (72.22)%	-	0.41	2(66.6%)	69(71.13%)	-	0.86
_ +ve_	4(40.0%)	25 (27.7%)			1(3.33%)	28(28.86%)		
EEG								
_ -ve_	4(40.0%)	54(60.0%)			1(3.33%)	57(58.7%)		
_ +ve_	6(60.0%)	36(40.0%)	-	0.224	2(66.6%)	40(41.23%)	-	0.37
Delivery history								
_ -ve_	7 (70.0%)	67(74.44%)	-	0.761	2(66.6%)	72(74.23%)		
_ +ve_	3(30.0%)	23(25.5%)			1(3.33%)	25(25.77%)		0.76
GTT symptoms								
_ -ve_	6(60.0%)	39(39.0%)			1(3.33%)	44(45.36%)	-	0.68
_ +ve_	4(40.0%)	51(51.0%)	-	0.58	2(66.6%)	53(54.63%)		
Sleeping								
_ -ve_	5(50.0%)	62(68.8%)	-	0.12	3(100%)	64(64.0%)	-	0.217
_ +ve_	5(50.0%)	28(31.11#)			0	33(33.0%)		

^*^Significant difference at *P* value ≤ 0.05

There was a significant correlation between the first variation (rs360932) and the gender, CARS, and degree of disease in ASD children (*P* = 0.00, *P* = 0.001, and *P* = 0.01, respectively). Additionally, a significant difference was observed in the rs360932 variant between males and females (*P* = 0.000), whereas the GA genotype was more common in males (81.11%). Moreover, the rate of CARS was significantly associated with the GG genotype in rs360932 (45.9) compared with that of the GA genotype (38.66). It was found that the severity of disease was highly recorded in the GG genotype (70%) compared to the AG genotype (23.33%), while the mild degree of the disease represented 43.33% in the AG genotype and only 20% in the GG genotype patients. However, no statistically significant differences were found comparing the second variant, rs363598 with the clinical characteristics of ASD children (*P* value > 0.05).

## Discussion

Autism spectrum disorder (ASD) is a neurodevelopmental disorder with a biological background, the recorded incidence of ASD has shown a significant increase over the last three decades with unknown etiology till now [42–44]. A variety of variables, including genetic and environmental factors, may correlate with the development of ASD in children [44, 45]. For this reason, identifying the suitable genes responsible for ASD is challenging [46, 47].

The presented case–control study is the first to look at the relationship between the appearance of ASD in Egyptian children and two genetic variants in the GRIK1 gene, rs363598 T > C and intergenic rs360932 G > A.

A validated Tetra-primer ARMS-PCR method was utilized to determine the SNPs mentioned above instead of the conventional PCR methods. The tetra-primer ARMS-PCR method has the advantage of being a reliable and rapid method for the identification of small deletions or point mutations [48].

Herein, our represented population of ASD patients includes 77% males, while 23% females (male to female ratio is 3.5:1). ASD children have lower IQ than healthy children, and 42% of ASD children have affected families with psychological disease. The mean of clinically evaluated CARS (39.40 ± 6.25) divided the children into 41% mild, 31% moderate, and 28% severe ASD. Our findings indicated that the genotype distribution of rs360932 G > A showed a significant relationship with autistic children, with a significant association with the gender of ASD children, CARS, and the degree of the disease. Furthermore, there were no statistically significant differences compared to the second variant, rs363598.

It was reported that, ASD development is more associated with boys as males have minor threshold for brain dysfunction than females, causing higher incidence of autism in males [32–49]. For that, more severe brain damage would be essential to produce autism in girls, resulting in a more severely impaired autistic child [50, 51]. It was found that analyzing intellectual profiles in relation to age and gender is important for the clinical management of ASD among children [52, 53].

Much research has been conducted in various demographic groups and geographical areas [54], followed by several biochemical pathways to demonstrate the role of genes [55, 56]. Genetic studies of ASD include cytogenetic analysis, copy number variation (CNV) analysis, linkage and association studies, microarray analysis, and studies of synaptic genes [44]. A previous study explored the role of copy number variations (CNVs) in ASD. The identified genes are implicated in pathways associated with neurodevelopment and synaptic function, providing insights into the genetic architecture of ASD [57, 58].

Recently, a study utilizing the whole genome database in the MSSNG program identified approximately 61 genetic variants that impact the risk of developing ASD [59]. Several studies were performed to ascertain the function of kainate receptors (KARs) in many diseases, for example, ASD, ischemic brain damage, schizophrenia, and epilepsy disorder [60, 61]. KARs have a large distribution, both pre- and post-synaptically, on many brain cell types that participate in the regulation of neurotransmitter release, control of synaptic networking, increase in neuronal excitability, and regulation of both excitatory and inhibitory transmission [62].

The KARs, encoded by GRIK1–GRIK5 genes, form functional ion channels through combinations of five subunits, such as GluK1–GluK5 [63], where KAR modulates both GABA and glutamate release [64–66].

It is well known that the brain areas where the GRIK1-containing glutamate receptors (GluK) are expressed [67] are necessary for memory and learning [68]. Additionally, inflammation in the central nervous system (CNS) can be reduced by regulating glutamate receptor binding activity, -axonal damage and apoptosis [69]. The GRIK1 gene is located on chromosome 21q22.1 and includes 18 exons. A wide range of neurological conditions have been linked to common non-coding, intronic variations and de novo genetic changes carried in GluK subunit genes in humans [70].

A previous study conducted on Bangladeshi children found an association between the GRIK1 gene rs363598 T > C and the intergenic rs360932 G > A polymorphisms and ASD development. In addition, a substantial linkage was also revealed for both SNPs in both dominant and recessive models [15]. Similarly, our study detected a significant association between the rs360932 G > A variant and the ASD disorder. However, no significant difference was found in the rs363598 variant. ASD is currently classified as a heterogeneous disorder but a spectrum with varying levels of impact on an individual’s functioning and the etiology is still unknown with strong association of genetic susceptibility [43–71].

## Conclusion

To the best of our knowledge, the presented case–control study is the first to focus on the GRIK1 gene rs363598 T > C and the intergenic rs360932 G > A polymorphisms in ASD Egyptian children, utilizing the ARMS-PCR technique with tetra-primers. A significant association was identified for rs360932 with ASD. However, for a better understanding of the genetic risk factors contributing to ASD development, it is recommended to conduct larger-scale studies to accurately reflect the Egyptian population and the correlation of ASD with other genes.

Relevant limitations of this research include the small sample size and the study of the association of only two SNPs with ADS in Egyptian cases.

## Data Availability

Availability of data and materials. All data and materials are available from the corresponding author.

## References

[1] Tian J, Gao X, Yang L. Repetitive restricted behaviors in autism spectrum disorder: from mechanism to development of therapeutics. Front Neurosci. 2022;16:780407. 10.3389/fnins.2022.780407. 35310097 10.3389/fnins.2022.780407 PMC8924045

[2] Hirota T, King BH. Autism spectrum disorder: a review. JAMA. 2023;329:157. 10.1001/jama.2022.23661. 36625807 10.1001/jama.2022.23661

[3] Ghamari R, Tahmaseb M, Sarabi-Jamab A, Etesami S-A, Mohammadzadeh A, Alizadeh F, et al. Association of verbal and non-verbal theory of mind abilities with non-coding variants of OXTR in youth with autism spectrum disorder and typically developing individuals: a case-control study. BMC Psychiatry. 2024;24:30. 10.1186/s12888-023-05461-w. 38191308 10.1186/s12888-023-05461-w PMC10773038

[4] Okoye C, Obialo-Ibeawuchi CM, Obajeun OA, Sarwar S, Tawfik C, Waleed MS, et al. Early diagnosis of autism spectrum disorder: a review and analysis of the risks and benefits. Cureus. 2023. 10.7759/cureus.43226. Cited 9 Sep 2024. 10.7759/cureus.43226 PMC10491411 37692637

[5] Fombonne E, MacFarlane H, Salem AC. Epidemiological surveys of ASD: advances and remaining challenges. J Autism Dev Disord. 2021;51:4271–90. 10.1007/s10803-021-05005-9. 33864555 10.1007/s10803-021-05005-9

[6] Zeidan J, Fombonne E, Scorah J, Ibrahim A, Durkin MS, Saxena S, et al. Global prevalence of autism: a systematic review update. Autism Res. 2022;15:778–90. 10.1002/aur.2696. 35238171 10.1002/aur.2696 PMC9310578

[7] Yousef AM, Roshdy EH, Abdel Fattah NR, Said RM, Atia MM, Hafez EM, et al. Prevalence and risk factors of autism spectrum disorders in preschool children in Sharkia, Egypt: a community-based study. Middle East Curr Psychiatry. 2021;28:36. 10.1186/s43045-021-00114-8.

[8] Metwally AM, Abdallah AM, El-Din EMS, Zeid DA, Khadr Z, Elshaarawy GA, et al. Screening and determinant of suspected developmental delays among Egyptian preschool-aged children: a cross-sectional national community-based study. BMC Pediatr. 2023;23(1):521. 10.1186/s12887-023-04335-0. 37858055 10.1186/s12887-023-04335-0 PMC10585886

[9] Metwally AM, Salah El-Din EM, Sami SM, Abdelraouf ER, Sallam SF, Elsaeid A, et al. Mapping autism in Egypt: population-based insights into prevalence, risk determinants, and severity among children aged 1–12 years. Mol Autism. 2025;16(1):32. 10.1186/s13229-025-00665-1. 40442748 10.1186/s13229-025-00665-1 PMC12121136

[10] Hodges H, Fealko C, Soares N. Autism spectrum disorder: definition, epidemiology, causes, and clinical evaluation. Transl Pediatr. 2020;9:S55–65. 10.21037/tp.2019.09.09. 32206584 10.21037/tp.2019.09.09 PMC7082249

[11] Genovese A, Butler MG. Clinical assessment, genetics, and treatment approaches in Autism Spectrum Disorder (ASD). Int J Mol Sci. 2020;21(13):4726. 10.3390/ijms21134726. 32630718 10.3390/ijms21134726 PMC7369758

[12] De Rubeis S, Buxbaum JD. Genetics and genomics of autism spectrum disorder: embracing complexity. Hum Mol Genet. 2015;24:R24–31. 10.1093/hmg/ddv273. 26188008 10.1093/hmg/ddv273 PMC4675826

[13] Nahas LD, Datta A, Alsamman AM, Adly MH, Al-Dewik N, Sekaran K, et al. Genomic insights and advanced machine learning: characterizing autism spectrum disorder biomarkers and genetic interactions. Metab Brain Dis. 2023;39:29–42. 10.1007/s11011-023-01322-3. 38153584 10.1007/s11011-023-01322-3 PMC10799794

[14] Lin F, Li J, Wang Z, Zhang T, Lu T, Jiang M, et al. Replication of previous autism-GWAS hits suggests the association between NAA1, SORCS3, and GSDME and autism in the Han Chinese population. Heliyon. 2024;10:e23677. 10.1016/j.heliyon.2023.e23677. 38234914 10.1016/j.heliyon.2023.e23677 PMC10792458

[15] Aziz MdA, Akter T, Hussain MdS, Millat MdS, Uddin MS, Sajal Md, et al. Association of rs363598 and rs360932 polymorphisms with autism spectrum disorder in the Bangladeshi children. Meta Gene. 2020;25:100733. 10.1016/j.mgene.2020.100733.

[16] Diguet E, Fernagut PO, Normand E, Centelles L, Mulle C, Tison F. Experimental basis for the putative role of GluR6/kainate glutamate receptor subunit in Huntington’s disease natural history. Neurobiol Dis. 2004;15:667–75. 10.1016/j.nbd.2003.12.010. 15056475 10.1016/j.nbd.2003.12.010

[17] Mironova YS, Zhukova IA, Zhukova NG, Ivanova SA, Alifirova VM, Boiko AS, et al. Parkinson’s disease and polymorphisms of the glutamatergic system genes GRIN2A, SLC1A2, and GRIK4. Neurol Neuropsychiatr Psychosom. 2018;10(2):27–32. 10.14412/2074-2711-2018-2-27-32.

[18] Takenouchi T, Hashida N, Torii C, Kosaki R, Takahashi T, Kosaki K. 1p34.3 deletion involving GRIK3: further clinical implication of GRIK family glutamate receptors in the pathogenesis of developmental delay. Am J Med Genet A. 2014;164A(2):456–60. 10.1002/ajmg.a.36240. 24449200 10.1002/ajmg.a.36240

[19] Hu T-M, Wu C-L, Hsu S-H, Tsai H-Y, Cheng F-Y, Cheng M-C. Ultrarare loss-of-function mutations in the genes encoding the ionotropic glutamate receptors of kainate subtypes associated with schizophrenia disrupt the interaction with PSD95. JPM. 2022;12:783. 10.3390/jpm12050783. 35629206 10.3390/jpm12050783 PMC9144110

[20] Traynelis SF, Wollmuth LP, McBain CJ, Menniti FS, Vance KM, Ogden KK, et al. Glutamate Receptor Ion Channels: Structure, Regulation, and Function. Sibley D, editor. Pharmacol Rev. 2010;62:405–496. 10.1124/pr.109.002451. 20716669 10.1124/pr.109.002451 PMC2964903

[21] Kranzler HR, Gelernter J, Anton RF, Arias AJ, Herman A, Zhao H, et al. Association of markers in the 3′ region of the GluR5 kainate receptor subunit gene to alcohol dependence. Alcohol Clin Exp Res. 2009;33:925–30. 10.1111/j.1530-0277.2009.00913.x. 19320626 10.1111/j.1530-0277.2009.00913.x PMC2772659

[22] Izzi C, Barbon A, Kretz R, Sander T, Barlati S. Sequencing of the *GRIK1* gene in patients with juvenile absence epilepsy does not reveal mutations affecting receptor structure. Am J Med Genet. 2002;114:354–9. 10.1002/ajmg.10254. 11920863 10.1002/ajmg.10254

[23] Kranzler HR, Covault J, Feinn R, Armeli S, Tennen H, Arias AJ, et al. Topiramate treatment for heavy drinkers: moderation by a *GRIK1* polymorphism. AJP. 2014;171:445–52. 10.1176/appi.ajp.2013.13081014. 10.1176/appi.ajp.2013.13081014 PMC3997125 24525690

[24] Nisar S, Bhat AA, Masoodi T, Hashem S, Akhtar S, Ali TA, et al. Genetics of glutamate and its receptors in autism spectrum disorder. Mol Psychiatry. 2022;27(5):2380–92. 10.1038/s41380-022-01506-w. 35296811 10.1038/s41380-022-01506-w PMC9135628

[25] Lee PH, Anttila V, Won H, Feng Y-CA, Rosenthal J, Zhu Z, et al. Genomic relationships, novel loci, and pleiotropic mechanisms across eight psychiatric disorders. Cell. 2019;179:1469-1482.e11. 10.1016/j.cell.2019.11.020. 31835028 10.1016/j.cell.2019.11.020 PMC7077032

[26] Cross-Disorder Group of the Psychiatric Genomics Consortium. Identification of risk loci with shared effects on five major psychiatric disorders: a genome-wide analysis. Lancet. 2013;381:1371–9. 10.1016/S0140-6736(12)62129-1. 23453885 10.1016/S0140-6736(12)62129-1 PMC3714010

[27] González-Castejón M, Marín F, Soler-Rivas C, Reglero G, Visioli F, Rodríguez-Casado A. Functional non-synonymous polymorphisms prediction methods: current approaches and future developments. Curr Med Chem. 2011;18(33):5095–103. 10.2174/092986711797636081. 22050757 10.2174/092986711797636081

[28] Ratanatharathorn A, Koenen KC, Chibnik LB, Weisskopf MG, Rich-Edwards JW, Roberts AL. Polygenic risk for autism, attention-deficit hyperactivity disorder, schizophrenia, major depressive disorder, and neuroticism is associated with the experience of childhood abuse. Mol Psychiatry. 2021;26(5):1696–705. 10.1038/s41380-020-00996-w. 33483690 10.1038/s41380-020-00996-w PMC8164961

[29] Thapar A, Livingston LA, Eyre O, Riglin L. Practitioner review: Attention-deficit hyperactivity disorder and autism spectrum disorder – the importance of depression. Child Psychology Psychiatry. 2023;64:4–15. 10.1111/jcpp.13678. 10.1111/jcpp.13678 PMC10087979 35972029

[30] Garcia-Argibay M, Brikell I, Thapar A, Lichtenstein P, Lundström S, Demontis D, et al. Attention-deficit/hyperactivity disorder and major depressive disorder: evidence from multiple genetically informed designs. Biol Psychiatry. 2023;S0006322323014622. 10.1016/j.biopsych.2023.07.017. 10.1016/j.biopsych.2023.07.017 37562520

[31] Schopler E, Van Bourgondien ME, Wellman GJ, Love SR. Test Review. E. Schopler, M. E. Van Bourgondien, G. J. Wellman, & S. R. Love Childhood Autism Rating Scale (2nd ed). Los Angeles: Western Psychological Services; 2010.

[32] Seif Eldin A, Habib D, Noufal A, Farrag S, Bazaid K, Al-Sharbati M, et al. Use of M-CHAT for a multinational screening of young children with autism in the Arab countries. Int Rev Psychiatry. 2008;20:281–9. 18569180 10.1080/09540260801990324

[33] Tian P, Zhu X, Liu Z, Bian B, Jia F, Dou L, et al. Effects of vitamin D on brain function in preschool children with autism spectrum disorder: a resting-state functional MRI study. BMC Psychiatry. 2025;25(1):198. 10.1186/s12888-025-06534-8. 40033268 10.1186/s12888-025-06534-8 PMC11877728

[34] Santos CLD, Barreto II, Floriano I, Tristão LS, Silvinato A, Bernardo WM. Screening and diagnostic tools for autism spectrum disorder: systematic review and meta-analysis. Clinics. 2024;79:100323. 10.1016/j.clinsp.2023.100323. 38484581 10.1016/j.clinsp.2023.100323 PMC10951453

[35] Silva BBL, Xavier IALN, Lima RASC, Delgado I, Montenegro ACA. Analysis of scores in the Childhood Autism Rating Scale of children with autism spectrum disorder before and after intervention with the method – development of communication skills in autism. Rev CEFAC. 2024;26(5):e1624. 10.1590/1982-0216/20242651624.

[36] NCSS, LLC. PASS 2024 Power analysis and sample size software. Kaysville (UT): NCSS, LLC. 2024. Available from: https://www.ncss.com/software/pass/. Cited 2025 Sep 14.

[37] Carayol J, Schellenberg GD, Tores F, Hager J, Ziegler A, Dawson G. Assessing the impact of a combined analysis of four common low-risk genetic variants on autism risk. Mol Autism. 2010;1(1):4. 10.1186/2040-2392-1-4. 20678243 10.1186/2040-2392-1-4 PMC2907567

[38] Hong EP, Park JW. Sample size and statistical power calculation in genetic association studies. Genomics Inform. 2012;10(2):117–22. 10.5808/GI.2012.10.2.117. 23105939 10.5808/GI.2012.10.2.117 PMC3480678

[39] Mostafa GA, Shehab AA, Al-Ayadhi LY. The link between some alleles on human leukocyte antigen system and autism in children. J Neuroimmunol. 2013;255(15):70–4. 10.1016/j.jneuroim.2012.10.002. 23110937 10.1016/j.jneuroim.2012.10.002

[40] Metwally AM, Amer HA, Salama HI, Abd El Hady SI, Alam RR, Aboulghate A, et al. Egyptian patients’/guardians’ experiences and perception about clinical informed consent and its purpose: cross sectional study. PLoS ONE. 2021;16(6):e0252996. 34125842 10.1371/journal.pone.0252996 PMC8202917

[41] Ye S, Dhillon S, Ke X, Collins AR, Day IN. An efficient procedure for genotyping single nucleotide polymorphisms. Nucleic Acids Res. 2001;29:88e–8. 10.1093/nar/29.17.e88. 10.1093/nar/29.17.e88 PMC55900 11522844

[42] American Psychiatric Association, DSM-5 Task Force. Diagnostic and statistical manual of mental disorders: DSM-5™ (5th ed.). American Psychiatric Publishing, Inc. 2013. 10.1176/appi.books.9780890425596.

[43] Hansen SN, Schendel DE, Parner ET. Explaining the increase in the prevalence of autism spectrum disorders: the proportion attributable to changes in reporting practices. JAMA Pediatr. 2015;169:56–62. 25365033 10.1001/jamapediatrics.2014.1893

[44] Almandil NB, Alkuroud DN, AbdulAzeez S, AlSulaiman A, Elaissari A, Borgio JF. Environmental and genetic factors in autism spectrum disorders: special emphasis on data from Arabian studies. IJERPH. 2019;16:658. 10.3390/ijerph16040658. 30813406 10.3390/ijerph16040658 PMC6406800

[45] Catalá-López F, Hutton B, Page MJ, Driver JA, Ridao M, Alonso-Arroyo A, et al. Mortality in persons with Autism Spectrum Disorder or Attention-Deficit/Hyperactivity Disorder: a systematic review and meta-analysis. JAMA Pediatr. 2022;176:e216401. 10.1001/jamapediatrics.2021.6401. 35157020 10.1001/jamapediatrics.2021.6401 PMC8845021

[46] Bailey A, Le Couteur A, Gottesman I, Bolton P, Simonoff E, Yuzda E, et al. Autism as a strongly genetic disorder: evidence from a British twin study. Psychol Med. 1995;25:63–77. 10.1017/S0033291700028099. 7792363 10.1017/s0033291700028099

[47] Huguet G, Benabou M, Bourgeron T. The Genetics of Autism Spectrum Disorders. In: Sassone-Corsi P, Christen Y, editors. Time for Metabolism and Hormones. Cham: Springer International Publishing; 2016. pp. 101–129. 10.1007/978-3-319-27069-2_11. 28892342

[48] Linjawi SA, Al-gaithy ZK, Sindi S, Hamdi N, Linjawi A, Alrofidi A. TETRA-PRIMER ARMS-PCR as an efficient alternative for snps detection in molecular diagnostic: a comparison study. European J Pharma Med Res. 2019;2 (13):91–96.

[49] El-Hossiny RM, El Baz FM, Abdel Aziz EA, Abbas AA, Abdel Mageed RI, Abdel Raouf BA. HLA-DR4 gene expression in a sample of Egyptian autistic children and their mothers: is it a risk factor? Egypt J Pediatr Allergy Immunol. 2023;21(1):18–26. 10.21608/ejpa.2023.294364.

[50] Amaral DG, Schumann CM, Nordahl CW. Neuroanatomy of autism. Trends Neurosci. 2008;31(3):137–45. 18258309 10.1016/j.tins.2007.12.005

[51] Elfetoh DEA, Said Abdelhady NM, El-Hefnawy SM, Abd El Naby SA. RELN gene (rs 2229864) polymorphismas genetic risk factor in Egyptian children with autism spectrum disorders. Menoufia Med J. 2022;35(3):53. 10.4103/mmj.mmj_111_22.

[52] Al-Mamari W, Idris AB, Gabr A, Jalees S, Al-Jabri M, Abdulrahim R, et al. Intellectual profile of children with Autism Spectrum Disorder: identification of verbal and nonverbal subscales predicting intelligence quotient. Sultan Qaboos Univ Med J. 2021;21(3):386–93. 10.18295/squmj.4.2021.001. 34522403 10.18295/squmj.4.2021.001 PMC8407906

[53] Wolff N, Stroth S, Kamp-Becker I, Roepke S, Roessner V. Autism spectrum disorder and IQ - a complex interplay. Front Psychiatry. 2022;18(13):856084. 10.3389/fpsyt.2022.856084. 10.3389/fpsyt.2022.856084 PMC9058071 35509885

[54] Baxter AJ, Brugha TS, Erskine HE, Scheurer RW, Vos T, Scott JG. The epidemiology and global burden of autism spectrum disorders. Psychol Med. 2015;45:601–13. 10.1017/S003329171400172X. 25108395 10.1017/S003329171400172X

[55] Luck AN, Bobst CE, Kaltashov IA, Mason AB. Human serum transferrin: is there a link among autism, high oxalate levels, and iron deficiency anemia? Biochemistry. 2013;52:8333–41. 10.1021/bi401190m. 24152109 10.1021/bi401190m PMC3887466

[56] Senarathne U, Indika N-L, Jezela-Stanek A, Ciara E, Frye R, Chen C, et al. Biochemical, genetic and clinical diagnostic approaches to autism-associated inherited metabolic disorders. Genes. 2023;14:803. 10.3390/genes14040803. 37107561 10.3390/genes14040803 PMC10138025

[57] Griswold AJ, Ma D, Cukier HN, Nations LD, Schmidt MA, Chung RH, et al. Evaluation of copy number variations reveals novel candidate genes in autism spectrum disorder-associated pathways. Hum Mol Genet. 2012;21:3513–23. 22543975 10.1093/hmg/dds164 PMC3392110

[58] Al-Beltagi M, Saeed NK, Bediwy AS, Bediwy EA, Elbeltagi R. Decoding the genetic landscape of autism: a comprehensive review. World J Clin Pediatr. 2024;13(3):98468. 10.5409/wjcp.v13.i3.98468. 39350903 10.5409/wjcp.v13.i3.98468 PMC11438927

[59] Havdahl A, Niarchou M, Starnawska A, Uddin M, Van Der Merwe C, Warrier V. Genetic contributions to autism spectrum disorder. Psychol Med. 2021;51:2260–73. 10.1017/S0033291721000192. 33634770 10.1017/S0033291721000192 PMC8477228

[60] Mulle C, Crépel V. Regulation and dysregulation of neuronal circuits by KARs. Neuropharmacology. 2021;197:108699. 10.1016/j.neuropharm.2021.108699. 34246686 10.1016/j.neuropharm.2021.108699

[61] Molnár E. Kainate receptors in brain function and disorders. Neuropharmacology. 2022;207:108946. 10.1016/j.neuropharm.2022.108946. 34999012 10.1016/j.neuropharm.2022.108946

[62] Contractor A, Mulle C, Swanson GT. Kainate receptors coming of age: milestones of two decades of research. Trends Neurosci. 2011;34:154–63. 10.1016/j.tins.2010.12.002. 21256604 10.1016/j.tins.2010.12.002 PMC3051042

[63] Hansen KB, Wollmuth LP, Bowie D, Furukawa H, Menniti FS, Sobolevsky AI, et al. Structure, function, and pharmacology of glutamate receptor ion channels. Barker E, editor. Pharmacol Rev. 2021;73:1469–1658. 10.1124/pharmrev.120.000131. 10.1124/pharmrev.120.000131 PMC8626789 34753794

[64] Losada-Ruiz P, Falcón-Moya R, Rodríguez-Moreno A. Kainate receptors modulating glutamate release in the cerebellum. In: Uçar A, editor. Biogenic Amines in Neurotransmission and Human Disease. IntechOpen; 2019. 10.5772/intechopen.87984.

[65] Falcón-Moya R, Rodríguez-Moreno A. Metabotropic actions of kainate receptors modulating glutamate release. Neuropharmacology. 2021;197:108696. 10.1016/j.neuropharm.2021.108696. 34274351 10.1016/j.neuropharm.2021.108696

[66] Negrete-Díaz JV, Falcón-Moya R, Rodríguez-Moreno A. Kainate receptors: from synaptic activity to disease. FEBS J. 2022;289:5074–88. 10.1111/febs.16081. 34143566 10.1111/febs.16081

[67] Youn D, Gerber G, Sather WA. Ionotropic glutamate receptors and voltage-gated Ca ^2+^ channels in long-term potentiation of spinal dorsal horn synapses and pain hypersensitivity. Neural Plast. 2013;2013:1–19. 10.1155/2013/654257. 10.1155/2013/654257 PMC3808892 24224102

[68] Jane DE, Lodge D, Collingridge GL. Kainate receptors: pharmacology, function and therapeutic potential. Neuropharmacology. 2009;56:90–113. 10.1016/j.neuropharm.2008.08.023. 18793656 10.1016/j.neuropharm.2008.08.023

[69] Bolton C, Paul C. Glutamate receptors in neuroinflammatory demyelinating disease. Mediators Inflamm. 2006;2006:093684. 10.1155/MI/2006/93684. 10.1155/MI/2006/93684 PMC1592583 16883070

[70] Koromina M, Flitton M, Blockley A, Mellor IR, Knight HM. Damaging coding variants within kainate receptor channel genes are enriched in individuals with schizophrenia, autism and intellectual disabilities. Sci Rep. 2019;9:19215. 10.1038/s41598-019-55635-4. 31844109 10.1038/s41598-019-55635-4 PMC6915710

[71] Wang S, Sun Z, Martinez-Tejada LA, Yoshimura N. Comparison of autism spectrum disorder subtypes based on functional and structural factors. Front Neurosci. 2024;4(18):1440222. 10.3389/fnins.2024.1440222.eCollection. 10.3389/fnins.2024.1440222 PMC11486766 39429701

